# Cutting-edge approach for Alzheimer’s disease detection in the early stages: an overview

**DOI:** 10.1097/JS9.0000000000000177

**Published:** 2023-03-24

**Authors:** Ruhul Amin, Biplab K. Dey, Faruk Alam, Talha B. Emran

**Affiliations:** a Faculty of Pharmaceutical Science, Assam Down Town University, Panikhaiti, Guwahati, Assam, India; b Department of Pharmacy, BGC Trust University Bangladesh, Chittagong; c Department of Pharmacy, Faculty of Allied Health Sciences, Daffodil International University, Dhaka, Bangladesh

Highlights

- Alzheimer’s disease (AD) is the leading cause of dementia.
- Selective memory impairment is a hallmark of AD.
- A novel blood test may identify the underlying toxin in AD.
- Amyloid-beta plaques outside of cells in the brain could not be harmful on their own.

Alzheimer’s disease (AD) is the leading cause of dementia and a neurodegenerative ailment whose aetiology and pathophysiology are poorly understood. Selective memory impairment is a hallmark of AD and one of the first detectable symptoms in certain patients. Treatments exist to help with symptoms, but there is presently no cure, and the condition will worsen for all people eventually^1^.

A novel blood test may identify the underlying toxin in AD, years before the onset of memory loss and cognitive decline. AD molecular pathology begins early on with the creation of toxic amyloid-peptide-beta (Aβ) oligomers^2^. Potentially elevated levels of phosphorylated tau181 (p-tau181) in the plasma are also associated with AD^3^. Impaired neural signalling, neuroinflammation, tau phosphorylation, and neurodegeneration are only some of the downstream impacts of these oligomers, and it is believed that they begin 10–20 years before the onset of symptoms^4^.

However, new investigations have indicated that (Aβ) plaques are present in the brains of only about a third of Alzheimer’s patients, and that they are occasionally present in the brains of persons who exhibit no cognitive problems^5^.

The Aβ plaques outside of cells in the brain could not be harmful on their own, but might be the result of other, more elusive toxins. The intricacies are still being worked out, but the concept has inspired scientists at the University of Washington to develop a very precise soluble oligomer binding assay (SOBA)^6^. Initially, 310 people’s blood plasma was used to test SOBA. The cognitive health of the subjects ranged from moderate impairment to full-blown AD. Blood plasma measurements of toxic Aβ oligomers were used by SOBA to identify all 53 people with Alzheimer’s, a diagnosis confirmed by autopsy. Also, 11 people in the control group had oligomers in their plasma tested by SOBA^7^. In retrospect, we know that 10 of the individuals had moderate cognitive impairment or Alzheimer’s.

SOBA has the potential to be modified in the future to detect early signs of Parkinson’s disease, type II diabetes, and Lewy body dementia, all of which have been linked to protein misfolding. There have been several attempts to detect AD biomarkers, with mixed results.

A blood screening that includes identifies A precursors indicated Alzheimer’s up to 30 years before the cognitive abnormalities ever started to manifest. Other researchers looked at the feasibility of using a novel blood testing tool called Simoa to assess tau concentrations and forecast the onset of AD^8^. More than 400 people’s blood was sampled by the team. They determined the quantity of p-tau181, a variant of tau associated with AD, in plasma (the blood’s liquid component). P-tau181 in plasma was shown to be different between healthy subjects and those with Alzheimer’s pathology verified in autopsy. Additionally, the test may be able to distinguish between Alzheimer’s pathology and that of a group of uncommon neurodegenerative disorders known as frontotemporal lobar degeneration. Results from the spinal fluid p-tau181 test and the positron emission tomography brain scan for Aβ protein were similar to those from the plasma p-tau181 test, both of which are considered to be reliable biomarkers for AD^8^,^9^.

Now commonly used in clinical practice are blood tests screening for genes associated with AD. However, these tests are not very good at predicting who will get Alzheimer’s. With the use of a blood test, we could quickly screen a broader and more diversified pool of potential study participants. Improvements in early diagnosis of AD are a consequence of promising research such as SOBA and Simoa.

## Ethical approval

Not applicable.

## Sources of funding

No funding was received.

## Author contribution

R.A.: conceptualization, data curation, writing – original draft preparation, and writing – reviewing and editing. B.K.D. and F.A.: data curation, writing – original draft preparation, and writing – reviewing and editing. T.B.E.: writing – reviewing and editing, visualization, and supervision.

## Conflicts of interest disclosure

The authors declare that they have no conflicts of interest.

## Research registration unique identifying number (UIN)

None.

## Guarantor

T.B. Emran, PhD, Associate Professor, Department of Pharmacy, BGC Trust University Bangladesh, Chittagong 4381, Bangladesh. Tel: +880 303 356 193, fax: +880 312 550 224. https://orcid.org/0000-0003-3188-2272.

## Data availability

The data in this correspondence article is not sensitive in nature and is accessible in the public domain. The data is, therefore, available and not of a confidential nature.

## Provenance and peer review

Not commissioned, internally peer-reviewed.

## References

[1] Zhang C Zhang Y Lv Y . Chromone-based monoamine oxidase B inhibitor with potential iron-chelating activity for the treatment of Alzheimer’s disease. J Enzyme Inhib Med Chem 2023;38:100–17. 36519319 10.1080/14756366.2022.2134358 PMC9762789

[2] Dafnis I Argyri L Chroni A . Amyloid-peptide β 42 enhances the oligomerization and neurotoxicity of apoE4: the C-terminal residues Leu279, Lys282 and Gln284 modulate the structural and functional properties of apoE4. Neuroscience 2018;394:144–55. 30367942 10.1016/j.neuroscience.2018.10.026

[3] Karikari TK Pascoal TA Ashton NJ . Blood phosphorylated tau 181 as a biomarker for Alzheimer’s disease: a diagnostic performance and prediction modelling study using data from four prospective cohorts. Lancet Neurol 2020;19:422–33. 32333900 10.1016/S1474-4422(20)30071-5

[4] Amin R Quispe C Docea AO . The role of Tumour Necrosis Factor in neuroinflammation associated with Parkinson’s disease and targeted therapies. Neurochem Int 2022;158:105376. 35667491 10.1016/j.neuint.2022.105376

[5] Neff RA Wang M Vatansever S . Molecular subtyping of Alzheimer’s disease using RNA sequencing data reveals novel mechanisms and targets. *Sci Adv* 2021;7:eabb5398. 33523961 10.1126/sciadv.abb5398 PMC7787497

[6] Urton J . New blood test can detect ‘toxic’ protein years before Alzheimer’s symptoms emerge, study shows. *UW News*; 2022. Accessed 5 December 2022.

[7] Shea D Colasurdo E Smith A . SOBA: development and testing of a soluble oligomer binding assay for detection of amyloidogenic toxic oligomers. Proc Natl Acad Sci 2022;119:e2213157119. 36490316 10.1073/pnas.2213157119 PMC9897489

[8] Thijssen EH La Joie R Wolf A . Diagnostic value of plasma phosphorylated tau181 in Alzheimer’s disease and frontotemporal lobar degeneration. Nat Med 2020;26:387–97. 32123386 10.1038/s41591-020-0762-2 PMC7101073

[9] Janelidze S Mattsson N Palmqvist S . Plasma P-tau181 in Alzheimer’s disease: relationship to other biomarkers, differential diagnosis, neuropathology and longitudinal progression to Alzheimer’s dementia. Nat Med 2020;26:379–86. 32123385 10.1038/s41591-020-0755-1

